# Comparison between Peritoneal Macrophage Activation by* Bougainvillea xbuttiana* Extract and LPS and/or Interleukins

**DOI:** 10.1155/2017/4602952

**Published:** 2017-11-27

**Authors:** Lluvia Arteaga Figueroa, Rodolfo Abarca-Vargas, Claudia García Alanis, Vera L. Petricevich

**Affiliations:** Facultad de Medicina de la Universidad Autónoma del Estado de Morelos, Calle Iztaccihuatl Esq. Leñeros, Col. Volcanes, 62350 Cuernavaca, MOR, Mexico

## Abstract

Activation of macrophages may be one of the possible approaches in modulating inflammation. We previously reported that* Bougainvillea xbuttiana* extract showed an immunomodulatory activity. Here we compare the activation of macrophages exposed to* B. xbuttiana* extract and compare it with the other treatments such as LPS, IL-4, and IL-10. The cytotoxic effect of extract on peritoneal macrophages was determined by the technique of violet crystal staining. To verify the activation of macrophages we used the tests of vacuolization, hydrogen peroxide production, and percentages of cellular expansion and phagocytosis. The levels of interleukins secreted by macrophages treated with the extract, LPS, and cytokines were determined by the biological assay for the determination of TNF levels and by ELISA for all other interleukins. NO levels were evaluated by colorimetric reactions using Griess reagent. Our results showed that* B. xbuttiana* extract induced (a) low cytotoxicity percentages, (b) increased vacuolization, hydrogen peroxide production and cell expansion and phagocytosis percentages, and (c) decreased production of TNF-*α*, IFN-*γ*, IL-1*β*, and IL-6 and potentiated production of IL-4, IL-10 and TGF-*β*. These results suggest that* B. xbuttiana *extract was able to activate the murine macrophages in a manner similar to those macrophages exposed to IL-4 and IL-10.

## 1. Introduction

The World Health Organization recognizes the value of medicinal plants in the primary care of millions of people and estimates that more than 80% of the world's population uses these resources as the primary source of attention to their health problems. In Mexico, the genera,* B. spectabilis*,* B. glabra,* and* B. buttiana*, are used for the cure of respiratory disorders, such as cough, asthma, bronchitis, influenza, and pertussis [1–3]. The extracts of bracts and leaves of the genus* Bougainvillea* have been mentioned for their hepatoprotective, analgesic, anti-inflammatory, antioxidant, and immunomodulatory activities [1, 4, 5].

Depending on the tissue in which the macrophages are present may exhibit morphological and phenotypic differences. They can express a wide variety of recognition receptors and have a very active metabolism and secrete a large number of molecules, such as proteolytic enzymes, and generate reactive oxygen species (ROS), nitrogen and metabolites derived from arachidonic acid, chemokines, and interleukins [6].

Activation of macrophages can be reflected in the plasticity and versatility of these cells in response to different signals that are known as macrophage polarization. Thus, to define the two extremes of macrophage activation, the term M1 response is represented by proinflammatory activation or “classical activation,” while the term M2 response is anti-inflammatory activation or “alternative activation” [7].

Classical activation involves the exposure of the macrophage to substances such as interferon-*γ* (IFN-*γ*) or lipopolysaccharide (LPS), which are considered potent inducers of this type of activation. Macrophages activated by the classical pathway show changes in gene expression, metabolic pathways, and their morphological and functional characteristics. These changes are responsible for increased phagocytic capacity, processing and presentation of antigens, lysis of tumor cells, reactive oxygen species, and lysosomal enzymes. The elimination of intracellular parasites and secretion of interleukin 1*β* (IL-1*β*), IL-6, IL-8, and tumor necrosis factor (TNF-*α*) increased major histocompatibility complex (MHC) class II, CD86, CD16, CD32, CD64, and the antigenic presentation of helper T cells type 1 (TH1). In general, this type of activation is related to inflammatory states; however, a persistent inflammatory process is often counterproductive because it tends to damage tissues and in such cases the immune system should develop anti-inflammatory mechanisms [7, 8].

Activation of the alternative pathway was shown to be triggered by IL-4, IL-10, IL-13, transforming growth type *β*-glucocorticoids (TGB-*β*), and ligand-type Toll 4 receptors (TLR 4). This activation is dependent on some aspects such as the concentration of the inducer and as the degree of cell differentiation. In alternatively activated macrophages, the endocytic and phagocytic capacities are high. Thus, macrophages activated by this pathway are able to protect circulating tissues from prolonged inflammatory processes or from aggressive immune responses inducing tissue repair and/or modeling [8].

Activation of macrophages, by the classical and alternative pathway, is a complex process and finely regulated by a series of morphological and biochemical modifications that culminate in the increase of cellular capacity to exert its physiological functions [17, 18]. Macrophages differ in morphological form and functional status depending on each tissue and constitute 10 to 15% of the cells in quiescent conditions.

In* in vivo* homeostatic conditions, tissue-resident macrophages also exhibit a variable polarization state that is determined by the tissue microenvironment. The polarization of M1 macrophages predominates in the early stages of the inflammatory response, when high cytotoxic activity is detrimental to the tissue [7, 8, 17]. In other stages, the number of macrophages of the M2 phenotype increases in order to promote resolution of inflammation, limiting the Th1 type response and promoting Th2-cell recruitment. In general, it is accepted that M1-to-M2 repolarization occurs during inflammation [7, 8, 17, 18].

The imbalance of the M1/M2 ratio or an inappropriate change in macrophage polarization in inflamed tissues results in chronic inflammation, such as during the development of tumors, autoimmune diseases, insulin resistance, or cardiovascular disease associated with obesity [18, 19]. There are several endogenous compounds that participate in the inflammatory response and are known as mediators of this process. The most important functions of nitric oxide (NO) in the body are vascular tone modulator, central and peripheral neurotransmitter, platelet aggregation, and immune. In biological systems, nitric oxide decomposes into nitrites and nitrates and has been associated with different physiological conditions [19–21]. Other mediators such as the cytokines are produced by different types of immune system cells and are classified into two groups: (a) one group of proinflammatory cytokines TNF-*α*, IL-1, IL-6, IL-8, and IL-12 and (b) another group of anti-inflammatory cytokines such as IL-4 and IL-10 [22, 23]. We have previously described the immunomodulatory effect of* B. xbuttiana* extract on peritoneal macrophages of CD1 strain mice. In order to give continuity to those studies, this work was designed to compare the route of activation of peritoneal macrophages treated with extract of* B. xbuttiana* with the activation of macrophages stimulated with LPS, interleukins 4 and 10.

## 2. Material and Methods

### 2.1. Chemicals

RPMI-1640 medium, dimethylformamide, sodium nitrate, Naphthylethylenediamine (NAP), fetal bovine serum (FBS), ethanol, lipopolysaccharide (LPS), and 2,2′-azino-bis(3-ethylbenzothiazoline-6-sulfonic acid) (ABTS) were purchased from Sigma Aldrich Chemical Co. (Toluca, Mexico). The monoclonal antibodies used in this assay were anti-mouse capture and biotin-labeled detection and the respective interleukins recombinants were obtained from DB Biosciences Pharmingen (EUA).

### 2.2. Animals

Female mice of the BALB/c strain with a body weight of 20–25 g were obtained from the Bioterio of Instituto Nacional de Salud Publica (Cuernavaca, Mexico). The animals were maintained under a light and dark cycle of 12 hours and temperature of 23°C, with free access to food and water. All animals tests that were carried out on the use of animals are approved by the Animal Care Committee, with registration 005/2016.

### 2.3. Extract

*Bougainvillea xbuttiana* ethanolic extract* (BxbE)* was prepared and standardized as described in patent application (MX343163B) [24].

### 2.4. Chromatography GC-MS

The analyses were performed according to the method described by Abarca-Vargas et al. 2016 [25].

### 2.5. Peritoneal Macrophages

Macrophages were carried out identical to the method described by Cohn and Benson (1965) [26]. Briefly, the animals were anesthetized to wash the peritoneal cavity of each mouse with RPMI-1640 medium and the cell suspension obtained was centrifuged at 500 rpm for 5 minutes, and the cell pellet was resuspended at a concentration of 1 × 10^6^ cells/mL in RPMI-1640 medium supplemented with 10% fetal bovine serum (FBS) and distributed in 96-well microplates. The microplates were incubated at 37°C and 5% CO_2_ for 2 hours, and the nonadherent cells were removed by withdrawing the culture medium, and the adherent cells were then considered as peritoneal macrophages, which were stimulated with different treatments as described in Table 1. The different groups of macrophage cultures were incubated at 37°C with 5% CO_2_ and at distinct times the supernatants were collected and stored at −20°C until mediator determinations.

### 2.6. Determination of Cytotoxicity

The peritoneal macrophages were obtained and submitted to treatments as previously described. After distinct times the supernatants were discarded and the cells were stained with a crystal violet solution. The absorbance was determined by using of 655 nm filter. Cytotoxicity percent was calculated by the following formula:

(1) %  Cytotoxicity=1Absorbancesample – Absorbancecontrol×100.

### 2.7. Assays to Determine the Functional Conditions of Peritoneal Macrophages

#### 2.7.1. Determination of Vacuolization

Peritoneal macrophages were collected as mentioned above and exposed to different treatments and incubated with RPMI-1640 medium plus 10% of FBS. At indicated times the percentage of vacuolization was determined [27]. The supernatants were removed and 100 *μ*L of the neutral red dye constituted by 0.05 g of neutral red in 100 mL of PBS were added and incubated for an additional 30 minutes at 37°C and 5% of CO_2_. The excess dye was extracted using 100 *μ*L of methanol. Absorbance was determined on a microplate reader with a 570 nm wavelength filter. The percentage of vacuolization was determined with the following formula:

(2) %  Vacuolization=AbsorbancesampleAbsorbancecontrol−1×100.

#### 2.7.2. Determination of Hydrogen Peroxide (H_2_O_2_)

Peritoneal macrophages obtained as described above were distributed in 96-well microplates, exposed at different stimulations, and maintained at 37°C and 5% CO_2_. Concentration of hydrogen peroxide was determined directly on the cells in accordance to the method described by Pick and Mizel (1981) [28]. At different time of incubation, the cells were exposed to 100 *μ*L of phenol red dye constituted by use of 0.1%, 140 mM NaCl, and 10 mM K_2_PO_4_, supplemented with 5.5 mM peroxidase. The cells were kept for 1 hour at 37°C and 5% CO_2_, and the 10 *μ*L of 0.1 M NaOH solution was added. The absorbance was determined on a microplate reader with a 620 nm filter. The results were expressed in *μ*M and determined by comparison with the standard curve with hydrogen peroxide.

#### 2.7.3. Cell Expansion Assay

The assay for cell expansion was carried out as reported by Arruda et al. (2004) [29]. The cell suspension was distributed in 6-well microplates with coverslips and added with a volume of 1.0 × 10^5^ cells/well. The culture cells were incubated at 37°C with an atmosphere of 5% CO_2_, for 2 hours, and after that the cell cultures were washed for removal of the nonadherent cells. Next, the above-mentioned treatments were added to the cultures and incubated under the same conditions. At different times the supernatant was collected and the coverslips were stained with violet crystal for 20 seconds. The expanded cells were determined under a microscope with a 40x magnification. The results were expressed as a percentage, according to the following formula: % Cell expansion = (Number of expanded cells/Number of total cells) × 100.

#### 2.7.4. Phagocytosis Assay

The phagocytosis assay was performed as the method reported by Zebedee et al. (1994) [30]. In brief, we describe the obtaining of opsonization of the yeast. Yeast from the strain* Saccharomyces cerevisiae* Z-37 Ferring was incubated with the mice serum for 20–30 minutes at room temperature. After 2 washes with PSB pH 7.4 the yeasts were resuspended in 1 mL of PBS and a multiplicity of infection (MOI) of 5 was used for all assays.

*Preparation of Macrophages.* Briefly, in a 6-well microplate, coverslips were placed and a volume of 1.0 × 10^5^ cells per well. After 2 hours of incubation at 37°C in atmosphere of 5% CO_2,_ culture cells were washed for removal of the nonadherents and stimulated with* BxbE *extract, LPS, IL-4, and IL-10. At an indicated time the cells were washed twice with PBS and the opsonized yeast suspensions were added at a MOI of 5. After incubation for 90 minutes at 37°C and 5% CO_2_ atmosphere the cultures were washed with PBS, and for cell attachment absolute methanol was added for 20 minutes at 25°C. Subsequently, the cells were stained with aqueous safranin for 35 seconds and the removal of excess dye was performed with distilled water. The phagocytic index was calculated with the following equation:

(3) $$\begin{array}{c} Phagocytic\;\;index=\frac{Number\;\;of\;\;cells\;\;with\;\;yeast}{Number\;\;of\;\;total\;\;cells}. \end{array}$$

### 2.8. Determination of Inflammatory Mediators

The mediators present in the samples of supernatants of peritoneal macrophages obtained and subjected to the different treatments as described above were determined by the ELISA method and by the biological assay.

#### 2.8.1. Determination of Cytokines by the ELISA Method

The determination of the cytokines was performed by the method described by Schumacher et al. (1998) [31]. Briefly, 96-well microplates were prepared with 2 *μ*g/mL anti-mouse capture antibody for 6 hours at room temperature. The free sites of each well were blocked by addition of 100 *μ*l of PBS consisting of 10% FBS and 3% serum albumin (BSA). After incubation for 2 hours at 25°C, 50 uL of the experimental and control samples was added, and the microplates were placed at 4°C for 18 hours. Subsequently, for each well 1 *μ*g/mL of the biotin-labeled detection antibody was added and the microplates were incubated at room temperature for 45 minutes. After a final wash the reaction was developed by addition of ABTS to each well. The reaction time was between 10 and 80 minutes and quenched by the addition of SDS/N,N-dimethylformamide and the absorbance was determined on the microplate reader using a 405 nm filter. The results were expressed in pg/10^6^ cells and the minimum levels detected were 1 pg and 10 pg for IL-4, IL-5, and other cytokines, respectively.

#### 2.8.2. Detection of Cytokines by Biological Method

The levels of TNF present in the supernatants of experimental and control cultures were titrated according to the method previously described by Ruff and Gifford (1980) [32]. The L-929 cells were cultured in RPMI-1640 medium plus 10% FBS, adjusted at a concentration of 3–5 × 10^6^ cells/mL and dispersed at 96-well microplates. The cultures were incubated at 37°C in atmosphere of 5% CO_2_ for 18 hours. After this time, the culture medium was removed and 50 *μ*L per well RPMI 1640 medium plus 10% SFB was added to each well. To the first row of the microplate 25 *μ*L of the experimental samples and/or control was added, which were tested in triplicate and 50 *μ*L of RPMI-1640 supplemented with actinomycin D (1.0 *μ*L/mL) was added and the plates were incubated for 18 hours at 37°C with 5% CO_2_. Subsequently, the culture medium was extracted and the remaining cells were stained with a solution constituted by 0.02% crystal violet in 20% methanol at 25°C for 15 minutes. The excess dye absorbed by the fixed cells was removed by washing with water and 100 *μ*L of methanol was added. The absorbance was determined directly on the plate using a microplate reader with a 620 nm filter. The cytotoxic percent was estimated using the following equation:

(4) $$\begin{array}{c} \%\;\;Cytotoxicity=\left[\;1–\left(\;\frac{{Absorbance}_{sample}}{{Absorbance}_{control}}\;\right)\;\right]\times 100. \end{array}$$

The results were expressed in pg/10^6^ cells and compared to the standard curve of recombinant TNF (Boehringer Mannheim, Germany).

### 2.9. Nitric Oxide (NO) Determination

The levels of NO^2−^ present in the supernatants of experimental and/or control macrophages were determined by combining 50 *μ*L of the sample supernatant with 50 *μ*L of Griess reagent that was prepared with 0.1% sulfanilamide, 1.0% Naphthylethylenediamine (NAP) (Sigma Chemical Co. St. Louis, USA), and 3% phosphoric acid [33]. The control and experimental supernatants were kept at room temperature for 10 minutes. Absorbance was stablished employing a microplate reader with a filter 540 nm. The results were demonstrated in *μ*M/10^6^ cells in comparison to an established curve of sodium nitrite. The minimum levels detected in the experimental conditions were 1 *μ*M/10^6^ cells.

### 2.10. Statistical Analysis

The results were expressed as the mean +/− standard deviation of 4 replicates per test. Statistical evaluations were performed by Student's *t*-test analysis and the values were considered significant (*p* < 0.05).

## 3. Results

### 3.1. *B. xbuttiana *Ethanolic Extract

The structures, retention times, molecular weight, and biological activities of the compounds present in the* BxbE *extract are described in Table 2. Among the 9 compounds present in the* Bougainvillea xbuttiana* extract, two such as 2-Propenoic acid, 3-(2-hydroxyphenyl)-, (E)- and *n*-hexadecanoic acid with a retention time of 11.3 and 19.4 minutes and a molecular weight of 164.2 and 256.4 g/mol, respectively, are compounds which according to the relevant literature exhibit antioxidant, antinociceptive, and anti-inflammatory activities. While 3-O-methyl-D-glucose, tetradecanoic acid, 1-nonadecene, isopropyl palmitate, 1,2-benzenedicarboxylic diisooctyl ester, and squalene have preservative, analgesic, antioxidant, and antinociceptive activities, respectively. Biological activity was not found for the compound diisooctyl maleate.

### 3.2. *B. xbuttiana* Extract Effect on the Viability of Peritoneal Macrophages

**Figure 1:**
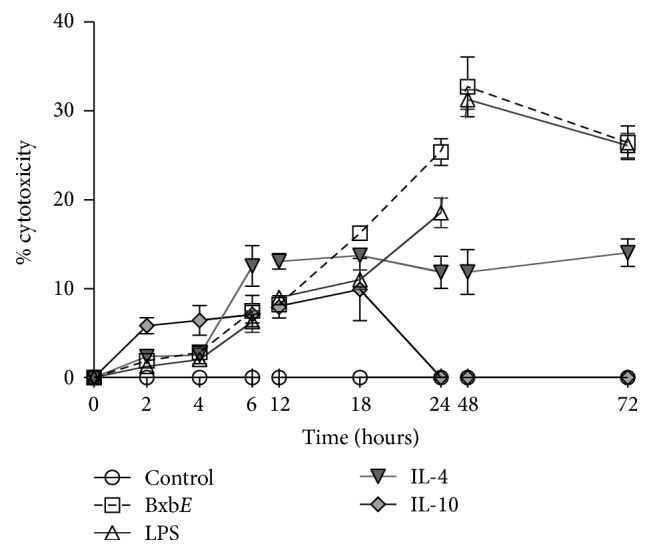
*Percentage of cytotoxicity*. Peritoneal macrophages were obtained, maintained, and submitted to different treatments as described in materials and methods. Each point represents the mean ± standard deviations of 4 different experiments.

The cytotoxic effect of* BxbE *extract on macrophage was estimated in cultures of cells exposed to extract and at different time intervals the percentage of cytotoxicity was determined. These percentages were compared with macrophage cultures stimulated with LPS, IL-4, and IL-10. As the concentration of extract increased, the number of viable cells reduced (data not shown). In this study,* BxbE *extract with 120 *μ*g/mL which is given better cell viability percentages has been used for further assays, since the cell viability is more than 70%.  Figure 1 shows the cytotoxic percentages of the macrophage culture groups subjected to the different stimulations. In the macrophages groups exposed to the extract of* B. xbuttiana* and LPS, the highest percentages of cytotoxicity were observed at 48 hours of exposure (27.31% and 32.72%). In groups of macrophages stimulated with IL-4 and IL-10, the highest percentages of cytotoxicity were observed at 12 and 4 hours, respectively. For these treatments at 48 hours, the percentages of cytotoxicity were significantly lower when compared to the macrophages groups treated with the* BxbE* extract (*p* < 0.001).

### 3.3. *B. xbuttiana* Extract Effect on the Macrophages Activation

To determine the activation status of macrophages cultures that were exposed to the extract, the following tests were performed: vacuolization, hydrogen peroxide production, percentages of cell spreading, and phagocytosis. The activation status of the macrophages treated with the extract was compared to the macrophages stimulated with LPS, IL-4, and IL-10.

#### 3.3.1. Kinetics of Formation of Vacuoles

**Figure 2:**
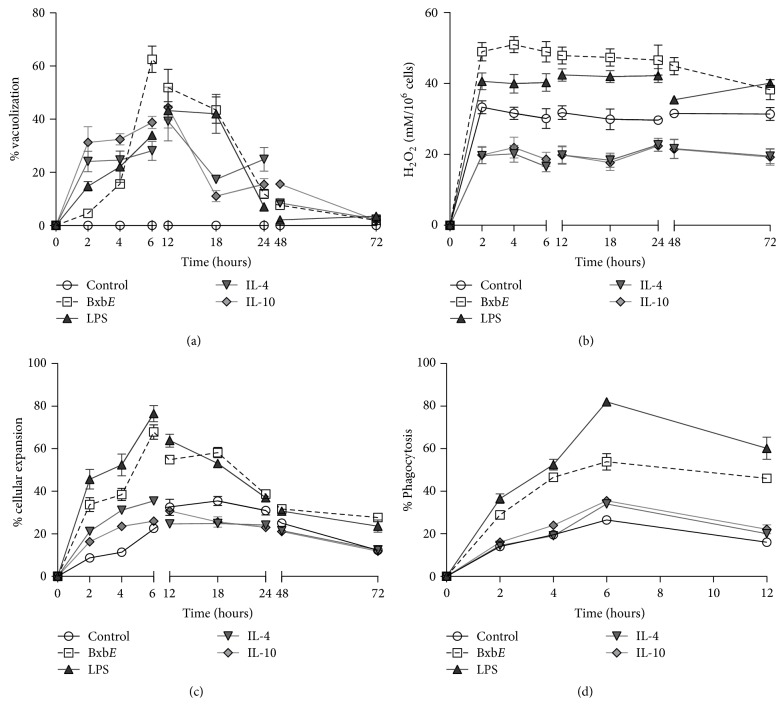
*Activation status*. (a) Vacuolization, (b) hydrogen peroxide production, (c) cell expansion, and (d) phagocytosis percentages. Macrophages were obtained and submitted to different treatments as described above. Each point represents the mean ± standard deviation of 4 different experiments.

In groups of peritoneal macrophages exposed to different treatments, the formation of vacuoles started at 2 hours (Figure 2(a)). The highest percentage of vacuoles formation in macrophages exposed to* BxbE* extract was observed at 6 hours with 62.59%, decreasing with time of exposure. The percentages of vacuolization in the macrophages stimulated with LPS, IL-4, and IL-10 were significantly lower when compared to cultures of macrophages exposed to* BxbE* for 6 hours (*p* < 0.05) (Figure 2(a)). The evaluation of the macrophages groups stimulated with LPS showed that the highest percentage of vacuoles formation was observed at 12 hours (43.22%), with subsequent decrease. For macrophage cultures stimulated with IL-4 and/or IL-10, the highest percentages of vacuolization were observed at 12 hours, with 39.25% and 44.43%, respectively (Figure 2(a)).

#### 3.3.2. Production of Hydrogen Peroxide

The production of hydrogen peroxide started at 2 hours of exposition (Figure 2(b)). Hydrogen peroxide levels produced in cultures of macrophages exposed to* BxbE* and LPS were observed at 2 until 48 hours, decaying with the time. The levels of hydrogen peroxide obtained in groups of macrophages exposed to IL-4 and IL-10 were significantly the lowest when compared with control cultures. Levels of hydrogen peroxide obtained in cultures of macrophages exposed to the extract were significantly higher when compared to those obtained from groups of macrophages treated with LPS and interleukins (*p* < 0.01) (Figure 2(b)).

#### 3.3.3. Expansion of Peritoneal Macrophages

For macrophages exposed to* BxbE* extract and LPS macrophages, the percentages of cell expansion were similar. For groups of macrophages stimulated with IL-4 and IL-10, the highest percentages of cell expansion were observed at 6 hours. The percentages of cell expansion in the macrophages groups exposed to* BxbE* extract and LPS were significantly higher when compared with those obtained from groups of macrophages exhibited to cytokines (*p* < 0.001) (Figure 2(c)).

#### 3.3.4. Dynamics of Phagocytosis in Peritoneal Macrophages

The highest percentages of phagocytosis in cultures of macrophages exposed to the different treatments were observed at 6 hours. In macrophages groups exposed to* BxbE* extract and LPS, the percentage of phagocytosis is similar until 4 hours. In contrast, in groups of macrophages exposed to* BxbE* extract and interleukins, the phagocytosis rates were significantly lower when compared to the percentage of phagocytosis percentages obtained in the macrophages groups exposed to LPS (*p* < 0.01) (Figure 2(d)).

### 3.4. Effect of the Extract* Bougainvillea xbuttiana* on the Production of Mediators

The effect of* B. xbuttiana* extract on the production of mediators was compared to LPS, IL-4, and IL-10 stimulated macrophages. For these assays the cell cultures were obtained, maintained, and treated as described above.

**Figure 3:**
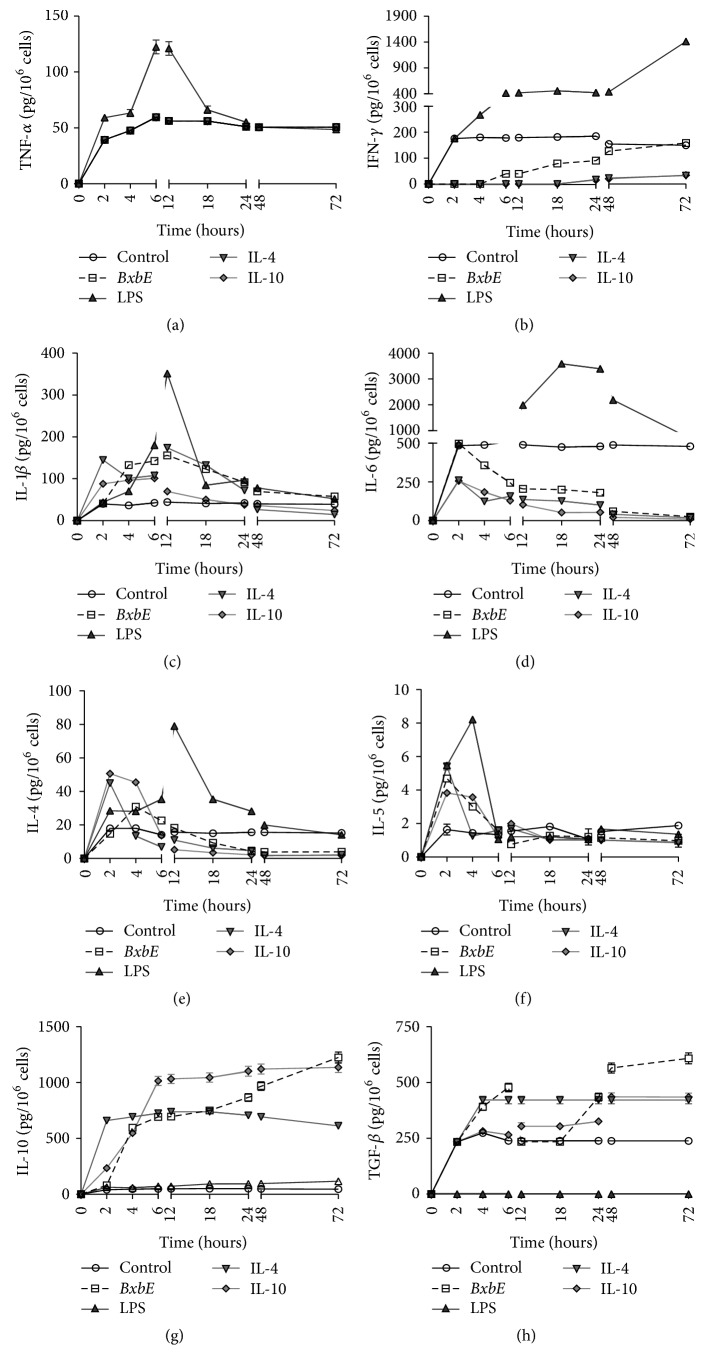
*Mediators production in peritoneal macrophages exposed to BxbE extract, LPS, and interleukins*. Peritoneal macrophages were obtained through peritoneal lavage and exposed to different treatments. At different time intervals, the supernatants were collected for the determination of interleukins levels as described above. Individual point represents the mean ± standard deviation of 4 different experiments. In all experiments, the standard deviations were less than 5% and therefore not observed.

*Production of TNF. *The production of TNF-*α* in groups of macrophages exposed to LPS begins to appear after 2 hours of exposure, reaching its maximum between 6 and 12 hours and decreasing subsequently. Similar production of TNF-*α* was observed in groups of macrophages exposed to* BxbE* extract, IL-4, and/or IL-10. The production of TNF-*α* recovered in cultures of peritoneal macrophages exposed to LPS was markedly higher in comparison to the production of TNF-*α* in control cultures or in macrophages exposed to the* BxbE* extract, IL-4, and IL-10 (*p* < 0.01) (Figure 3(a)).

*Production of IFN-γ.* IFN-*γ* levels present in the supernatants of the macrophages stimulated with LPS begin to appear from 2 hours of exposure and reach maximum production at 72 hours of exposure. The production of IFN-*γ* obtained in cultures of macrophages exposed to LPS was significantly higher when compared to those obtained from the cultures exposed to the* BxbE *extract. The production of IFN-*γ* in macrophages groups exposed to* BxbE *extract and to IL-4 and IL-10 was similar; it was also significantly lower when compared to the production of IFN-*γ* obtained in the control cultures (Figure 3(b)).

*Production of IL-1β.* In macrophages groups exposed to LPS, the highest levels of IL-1*β* were observed at 12 hours of exposure, decreasing subsequently. IL-1*β* production in groups of macrophages LPS stimulated was significantly higher when compared to that observed in the cultures exposed to the* BxbE *extract, IL-4, and IL-10 (*p* < 0.001). In groups of macrophages exposed to the extract and/or to interleukins the highest yields of IL-1*β* in the cultures exposed to the extract and/or to interleukins were obtained at 12 hours (Figure 3(c)).

*Production of IL-6.* The higher levels of IL-6 present in the cultures of macrophages exposed to LPS were observed at 18 and 24 hours, decreasing subsequently. IL-6 production in groups of macrophages LPS stimulated was significantly higher when compared to that obtained in cultures treated with the* BxbE *extract (*p* < 0.001). Similar levels of IL-6 were obtained from cultures of macrophages exposed to the* BxbE *extract and interleukin (Figure 3(d)).

*Production of IL-4.* The highest production of IL-4 in macrophages cultures exposed to LPS was at 12 hours of treatment. The levels of IL-4 obtained in cultures of macrophages LPS stimulated were significantly higher when compared to those obtained in macrophages exposed to the* BxbE *extract (*p* < 0.001). The highest levels of IL-4 in macrophages cultures exposed to IL-4 and IL-10 were observed within 2 hours of treatment. The IL-4 production in groups of macrophages exposed to the extract was significantly lower when compared to that obtained in cultures exposed to interleukins (*p* < 0.001) (Figure 3(e)).

*Production of IL-5.* The higher production of IL-5 in cultures of macrophages exposed to LPS was observed at 4 hours of treatment. The IL-5 production observed in groups of macrophages LPS stimulated was remarkably higher as to that obtained in macrophages groups treated with* BxbE* extract (*p* < 0.001). In contrast, in the macrophages cultures exposed to interleukins and/or the extract the higher levels of IL-5 were observed at 2 hours of exposure, decreasing subsequently (Figure 3(f)).

*Production of IL-10.* The levels of IL-10 present in the supernatants of the macrophages exposed to LPS were significantly lower when compared to those obtained in the cultures exposed to the* BxbE *extract and/or interleukins (*p* < 0.001). The production of IL-10 in macrophages cultures exposed to* BxbE* extract and to interleukins begins to appear at 2 hours and reaches a maximum at 72 hours of treatment. IL-10 production in cultures exposed to IL-10 for 6 up to 24 hours was significantly higher when compared to that obtained in cultures treated with the* BxbE *extract (*p* < 0.001) (Figure 3(g)).

*TGF-β Production.* In the supernatants of the macrophages exposed to LPS, TGF-*β* production was not observed. In contrast, the levels of TGF-*β* present in macrophages groups exposed to the* BxbE *extract were observed at two peaks, one at 6 hours and the other at 48 hours of treatment. The highest productions of TGF-*β* observed in macrophages cultures exposed to IL-4 and IL-10 were observed at 4 and 48 hours of exposure, respectively. The levels of TGF-*β* obtained in cultures of macrophages exposed to the* BxbE *extract were similar to those obtained in cultures of macrophages IL-4 stimulated up to 48 hours. The production of TGF-*β* in cultures of macrophages exposed to IL-10 was notably lower in comparison to those observed in groups of macrophages exposed to* BxbE* extract (*p* < 0.01) (Figure 3(h)).

### 3.5. Production of Nitric Oxide

**Figure 4:**
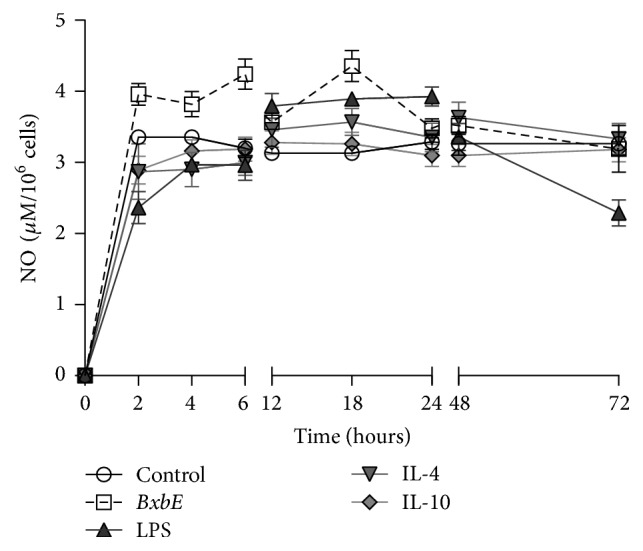
*NO production in peritoneal macrophages exposed to the BxbE extract, LPS, and interleukins*. Macrophages were obtained and subjected to different treatments. NO levels were determined through the Griess colorimetric assay. Each point represents the mean ± standard deviations of 6 different experiments (*p* < 0.01).

Figure 4 shows the levels of NO present in cultures of macrophages exposed to different treatments. NO production begins to appear within 2 hours of treatment. In the supernatants of macrophages exposed to* BxbE* extract the highest NO levels were observed at 18 hours and were significantly higher when compared to the NO levels present in macrophages exposed to LPS, IL-4, and IL-10 (*p* < 0.01).

### 3.6. Analysis of the Activation Pathway of the Extract of* Bougainvillea xbuttiana*

Table 3 describes a qualitative comparison of the mediators secreted by the macrophages treated with the* BxbE* extract, with the macrophages stimulated with LPS (M1), IL-4 (M2a), and IL-10 (M2c). Where the symbol of X represents little or no production of cytokines, the extract of* B. xbuttiana* presented a similarity of 33.3%, 55.55%, 33.3%, and 100% with the macrophages M1, M2a, M2b, and M2c, respectively. M2b macrophages are characterized by a phenotype very different from M2a and M2c. The stimulation with the* BxbE* extract was 100% similar to the macrophages IL-10 (M2c).

## 4. Discussion

The traditional use of the genus* Bougainvillea* is to treat diseases involving inflammatory processes such as cough, whooping cough, and pneumonia. Previous studies proved that this genus proved pharmacological activities are attributed as analgesics, antidiabetics, and anti-inflammatories among others [5]. All plants produce a huge diversity of secondary metabolites. This encouraged us to undertake additional studies and the involvement of such compounds in diseases. The results obtained from gas-mass chromatography showed the presence of 9 compounds. These compounds and their biological activities are described in Table 2.

In this study, we investigated the differential expression of murine macrophage M1 or M2 when exposed to the extract* B. xbuttiana*. For this, the route of activation of the peritoneal macrophages caused by exposure to the* BxbE* extract was compared to the macrophages stimulated with LPS (M1), IL-4 (M2a), and IL-10 (M2c). To establish the ideal conditions for the interaction of the* BxbE *extract with macrophages, cytotoxicity was evaluated. The results showed that the* BxbE* extract exerts a moderate effect on these phagocytic cells.

Activation of macrophages is an important event of the inflammatory process. The activation state of the macrophages exposed to the* BxbE *extract was evaluated by the formation of vacuoles, hydrogen peroxide production, cell spread, and phagocytosis which was compared to cultures of macrophages stimulated with LPS, IL-4, and IL-10. The effect of* BxbE* extract on macrophages showed morphological alterations, potentiated the formation of vacuoles, induced the production of hydrogen peroxide, and modified the percentages of phagocytosis and cellular expansion. These combined results provide evidence of cellular activation status. Previous studies have described that increased vacuole formation during stimulation is related to elevated exocytosis of inflammatory proteins. The activated macrophage presented several changes, such as an increase in pH, size, number of intracellular organelles, complexity, and acquisition of greater phagocytic capacity [34]. The particles ingested by phagocytic cells through receptor mechanisms involve a large mismatch of the cytoskeleton [34]. In addition, activated macrophages increase the expansion and adhesion to the endothelium. The cell expansion assays allow to observe the adhesion capacity of some substrates, such as glass or plastic, which are linked to the development of inflammatory reactions and phagocytosis, besides differentiating macrophages populations with different migratory behaviors, differentiation, and cell death [35].

In this study, the* BxbE* extract was able to induce low production of IFN-*γ*, IL-1*β*, IL-5, IL-6, and TNF-*α* and potentiate the production of IL-4, IL- 10, and TGF-*β*. Cytokines are a category of signaling molecules that mediate and regulate immunity, inflammation, hematopoiesis, and many other cellular processes, forming a cytokine network. In recent years, various investigations have demonstrated the use of extracts to prevent and treat inflammatory responses by inhibiting inflammatory cytokines such as TNF-*α*, IL-1*β*, and IL-6, and this has become an important area of investigation [1, 5]. There are several studies showing the role of IL-4 in increasing cytokine production in rat macrophages; these results may be explained by the fact that this interleukin is produced in activated macrophages and is capable of suppressing interleukins such as TNF-*α*, IL-5, and IFN-*γ* [6, 36].

Interleukin 6 is a cytokine that is involved in inflammatory processes, because its secretion can be induced by different stimuli likely LPS, TNF-*α*, and IL-1*β*. However, it may have a dual effect, since the initial proinflammatory response is controlled by immunoregulatory molecules such as specific inhibitors and soluble cytokine receptors [36]. The important anti-inflammatory cytokines are the groups of IL-1 receptor antagonist (IL-1ra), TGF-*β*, and interleukins 4, 6, 10, 11, and 13 [36]. However, in the present study the IL-6 levels present in macrophages exposed to IL-10, IL-4, and* BxbE* extract are low in the first 24 hours and by increasing exposure time the levels of IL-6 practically disappear.

The most important anti-inflammatory cytokine is IL-10. All studies indicate that macrophages activated classically, proinflammatory cytokine levels increase, over time, and elevated levels of anti-inflammatory interleukins begin to appear, as in the case of IL-10 [36]. Our results showed that macrophages exposed to* BxbE* extract induced significant IL-10 production. In addition, this interleukin is involved in different pathophysiologies [37]. Another anti-inflammatory cytokine and TGF-*β* when well balanced with IL-10 and IL-4 promotes M2 polarization [38]. Our results also showed high levels of TGF-*β* in groups of macrophages stimulated with* BxbE* extract. In contrast, when classically activated macrophages showed low levels of TGF-*β*, this can be explained by the fact that LPS acts negatively on the production of TGF-*β* [38].

Nitric oxide is a mediator directly related to the activation of macrophages and acts as a modulator of the immune system. In addition, in large amounts, it can inhibit the production of cytokines, but in small amounts they can induce them. NO produced by macrophages can inhibit the proliferation of T lymphocytes that occur during the cellular immune response [36]. In this work, it was demonstrated that macrophages exposed to* BxbE* extract produce high levels of NO.

The experimental conditions used in this study suggest that* Bougainvillea xbuttiana* extract showed the ability to activate murine macrophages in a manner similar to IL-4 and IL-10 exposed macrophages.

As shown in Table 3, a qualitative analysis of the mediators secreted by the stimulated macrophages. Based on the literature, classically activated macrophages (M1) that can be stimulated by LPS, IFN-*γ*, or TNF-*α* show appearance of Th1 type phenotype, which are proinflammatory effectors and include antibacterial activity, while macrophages (M2) alternately activated with three distinct phenotypes (M2a, b, and c) exhibit a Th2 type phenotype and they are involved in the decision of inflammation. The M2a phenotype is mainly induced by IL-4 and IL-13; these macrophages regulate the different components of the IL-1 system, which allows counteracting of its proinflammatory actions. In addition, they stimulate Arg1 expression in vitro, extinguishing the substrate and inhibiting the release of NO and IL-10. M2b macrophages can be induced by exposure to immune complexes; CD64 agonists opsonized with IgG as erythrocytes are characterized by a phenotype very distinct from M2a and M2c. These two types of macrophages have high IL-10 and small IL-12 expression, produce proinflammatory cytokines such as TNF-*α*, IL-1*β*, and IL-6, and do not express Arg1 and regulate CD86 positively. M2c macrophages are induced by IL-10 and glucocorticoids; IL-10 inhibits the production of proinflammatory cytokines, release of NO and ROS, and antimicrobial activities of macrophages. Furthering the mechanisms that define these phenotypes, it will be possible to better understand the influence and role of the alternatively activated macrophages in the immune responses regulated by them and in the pathogenesis of different diseases associated with the development of this group of macrophages [34].

## 5. Conclusion

Phenolic compounds have received much attention in recent years due to the various beneficial effects observed. Because of the antioxidant and anti-inflammatory activities, they have become important compounds with promising therapeutic potential. Our results showed that the production of interleukins in groups of macrophages exposed to the* BxbE* extract was similar to the profile of interleukins obtained in cultures exposed to IL-4 and IL-10. The combined results suggest that the* BxbE* extract can be able to alternatively activate the macrophages. Further studies are needed to understand the precise molecular mechanisms of* BxbE* extract on macrophage activation.

## Figures and Tables

**Table 1:** Scheme of stimulation of macrophages.

Macrophage stimulation	Terminology
Control macrophage	Control
Macrophage exposed to 120 *μ*g/100 *μ*L of *BxbE* extract	*BxbE*
Macrophage exposed to 10 *μ*g/100 *μ*L of LPS	LPS
Macrophage exposed to 10 ng/100 *μ*L of IL-4	IL-4
Macrophage exposed to 10 ng/100 *μ*L of IL-10	IL-10

**Table 2:** Description of the compounds found in the extract of *Bougainvillea xbuttiana*.

Compound	Chemical group	RT	GC	PM	Biological activity
		min	area%	g/Mol	
2-Propenoic acid, 3-(2-hydroxyphenyl)-, (E)-	Phenolic compound	11.3	11.24	164.2	Antioxidant [9].
3-O-Methyl-d-glucose	Carbohydrate	15.5	11.19	194.2	Preservative [10].
Tetradecanoic acid	Fatty acid	17.4	10.76	228.4	Larvicidal and repellent activity [11].
1-Nonadecene	Alkene	17.7	10.72	266.5	Antibacterial, antitubercular, and cytotoxic activities [12]
n-hexadecanoic acid	Fatty acid	19.4	11.51	256.4	Anti-inflammatory, antioxidant, and antinociceptive [13].
Isopropyl palmitate	Fatty acid	20.1	10.98	198.5	Antioxidant [14].
Diisooctyl maleate	Ester	21.9	11.26	340.5	No biological activity reported.
1,2-Benzenedicarboxylic acid, diisooctyl ester	Ester	25.7	11.23	390.6	Antifungal [15].
Squalene	Terpene	29.9	11.07	410.7	Antioxidant, antitumoral, and antifungal [16].

**Table 3:** Qualitative analysis of the mediators secreted by stimulated macrophages.

Stimulation	TNF-*α*	IFN-*γ*	IL-1*β*	IL-4	IL-5	IL-6	IL-10	TGF-*β*	NO
Classical route									
M1 exposed to LPS	✓	✓	✓	✓	✓	✓	X	X	X
Alternative route									
M2a	X	X	*✓*	*✓*	*✓*	*✓*	*✓*	*✓*	X
M2b	*✓*	*✓*	*✓*	X	X	*✓*	*✓*	*✓*	X
M2c	X	X	*✓*	*✓*	*✓*	X	*✓*	*✓*	*✓*
Ethanolic extract *Bougainvillea xbuttiana*	X	X	✓	✓	✓	X	✓	✓	✓

✓ (production) X (little or no production).

## Abbreviations

IL: Interleukin
IFN-*γ*: Interferon-gamma
LPS: Lipopolysaccharide
TGF-*β*: Transforming growth factor-beta
TNF-*α*: Tumor necrosis factor-alpha.
